# Temporal changes in *Plasmodium falciparum* anti-malarial drug sensitivity *in vitro* and resistance-associated genetic mutations in isolates from Papua New Guinea

**DOI:** 10.1186/s12936-015-0560-3

**Published:** 2015-01-28

**Authors:** Tamarah Koleala, Stephan Karl, Moses Laman, Brioni R Moore, John Benjamin, Celine Barnadas, Leanne J Robinson, Johanna H Kattenberg, Sarah Javati, Rina PM Wong, Anna Rosanas-Urgell, Inoni Betuela, Peter M Siba, Ivo Mueller, Timothy ME Davis

**Affiliations:** Papua New Guinea Institute of Medical Research, Madang, Papua New Guinea; School of Medicine and Pharmacology, University of Western Australia, Fremantle Hospital, PO Box 480, Fremantle, 6959 WA Australia; Infection and Immunity Division, Walter and Eliza Hall Institute, Parkville, VIC Australia; Department of Medical Biology, University of Melbourne, Melbourne, VIC Australia; Institute of Tropical Medicine, Antwerp, Belgium; Center de Recerca en Salut Internacional de Barcelona (CRESIB), Barcelona, Spain

**Keywords:** Malaria, *Plasmodium falciparum*, *in vitro* drug susceptibility, Resistance mutations

## Abstract

**Background:**

In northern Papua New Guinea (PNG), most *Plasmodium falciparum* isolates proved resistant to chloroquine (CQ) *in vitro* between 2005 and 2007, and there was near-fixation of *pfcrt* K76T, *pfdhfr* C59R/S108N and *pfmdr1* N86Y. To determine whether the subsequent introduction of artemisinin combination therapy (ACT) and reduced CQ-sulphadoxine-pyrimethamine pressure had attenuated parasite drug susceptibility and resistance-associated mutations, these parameters were re-assessed between 2011 and 2013.

**Methods:**

A validated fluorescence-based assay was used to assess growth inhibition of 52 *P. falciparum* isolates from children in a clinical trial in Madang Province. Responses to CQ, lumefantrine, piperaquine, naphthoquine, pyronaridine, artesunate, dihydroartemisinin, artemether were assessed. Molecular resistance markers were detected using a multiplex PCR ligase detection reaction fluorescent microsphere assay.

**Results:**

CQ resistance (*in vitro* concentration required for 50% parasite growth inhibition (IC_50_) >100 nM) was present in 19% of isolates. All piperaquine and naphthoquine IC_50_s were <100 nM and those for lumefantrine, pyronaridine and the artemisinin derivatives were in low nM ranges. Factor analysis of IC_50_s showed three groupings (lumefantrine; CQ, piperaquine, naphthoquine; pyronaridine, dihydroartemisinin, artemether, artesunate). Most isolates (96%) were monoclonal *pfcrt* K76T (SVMNT) mutants and most (86%) contained *pfmdr1* N86Y (YYSND). No wild-type *pfdhfr* was found but most isolates contained wild-type (SAKAA) *pfdhps*. Compared with 2005–2007, the geometric mean (95% CI) CQ IC_50_ was lower (87 (71–107) *vs* 167 (141–197) nM) and there had been no change in the prevalence of *pfcrt* K76T or *pfmdr1* mutations. There were fewer isolates of the *pfdhps* (SAKAA) wild-type (60 *vs* 100%) and *pfdhfr* mutations persisted.

**Conclusions:**

Reflecting less drug pressure, *in vitro* CQ sensitivity appears to be improving in Madang Province despite continued near-fixation of *pfcrt* K76T and *pfmdr1* mutations. Temporal changes in IC_50_s for other anti-malarial drugs were inconsistent but susceptibility was preserved. Retention or increases in *pfdhfr* and *pfdhps* mutations reflect continued use of sulphadoxine-pyrimethamine in the study area including through paediatric intermittent preventive treatment. The susceptibility of local isolates to lumefantrine may be unrelated to those of other ACT partner drugs.

**Trial registration:**

Australian New Zealand Clinical Trials Registry ACTRN12610000913077.

## Background

Resistance of *Plasmodium falciparum* to anti-malarial drugs in Papua New Guinea (PNG) began with chloroquine (CQ) in the 1970s [1] and has since extended to amodiaquine [2] and sulphadoxine-pyrimethamine (SP) [3]. Because of this trend, together with efficacy data from a large-scale, multi-arm, treatment trial conducted in coastal PNG from 2005 to 2007 [4] and World Health Organization management guidelines at the time [5], artemisinin combination therapy (ACT) was introduced nationally as recommended therapy for uncomplicated malaria in 2010 [6]. Artemether (AM) plus lumefantrine (LM) is currently first-line and dihydroartemisinin (DHA) plus piperaquine (PQ) second-line treatment in PNG, but artemisinin plus naphthoquine (NQ) is also available in the private sector [7]. Resistance to artemisinin derivatives has, however, emerged in recent years in Southeast Asia [8], and is a concern for countries such as PNG in which ACT is now widely used.

Regular testing using economical, robust and sensitive *in vitro* anti-malarial drug susceptibility assays is an integral part of the surveillance for parasite resistance [9]. Of the different methods currently available, those based on fluorescence measurements of parasite growth using inexpensive intercalating DNA stains such as Sybr Green and Pico Green have proved efficient and inexpensive without loss of sensitivity [10,11]. Additional insight into mechanisms of resistance is provided by detection of single nucleotide polymorphisms in parasite genes determining drug response [12], including mutations in the *P. falciparum* CQ transporter (*pfcrt*), multidrug resistance 1 (*pfmdr1*), dihydrofolate reductase (*pfdhfr*), and dihydropteroate synthetase (*pfdhps*) genes.

The most recent parasite drug resistance data from PNG were collected as part of the comparative intervention trial conducted in coastal Madang and East Sepik Provinces between 2005 and 2007 [13,14]. Most of the isolates tested proved resistant to CQ *in vitro* but not to other ACT partner drugs or to the artemisinin derivatives themselves [13]. Consistent with this finding and previous heavy 4-aminoquinoline/SP use, there was near-fixation of *pfcrt* K76T, *pfdhfr* C59R and S108N, and *pfmdr1* N86Y alleles, while multiple mutations were frequent [14].

To determine whether there has been any recent change in *P. falciparum* drug resistance in the north coastal PNG area, the *in vitro* susceptibility of local *P. falciparum* isolates collected between 2011 and 2013 to artemisinin derivatives and ACT partner drugs were re-assessed, and the prevalence of drug resistance markers in the same parasite strains re-examined.

## Methods

### Study sites, patients and ethical approval

Venous blood samples were obtained from 52 children aged six months to five years with an uncomplicated *P. falciparum* mono-infection at a parasitaemia >0.5% who were recruited at Mugil (n = 43) and Alexishafen (n = 9) health centres in Madang Province to a randomized, comparative, efficacy trial of the ACT AM-LM and artemisinin-NQ (Australian New Zealand Clinical Trials Registry ACTRN12610000913077) [15]. The study received ethical approval from the Medical Research Advisory Committee of the PNG Department of Health (MRAC #10.39). In all cases, informed consent was obtained from the parents or legal guardians before recruitment and blood sampling.

### Drug susceptibility assays

A Sybr Green fluorescence assay was used to assess drug susceptibility. All assays were carried out at the PNG Institute of Medical Research in Madang. The methodology used, a modified version of that first described by Smilkstein *et al.* [11], has been previously validated against tritium hypoxanthine incorporation, *Pf* lactate dehydrogenase (*Pf*LDH), light microscopic schizont maturation, and flow cytometry-based drug susceptibility assays using the laboratory-adapted parasite strains 3D7, E8B and W2 [16]. For the present series of experiments, the 3D7 strain was used as reference with a mean CQ *in vitro* concentration required for 50% parasite growth inhibition (IC_50_) value of 14.3 nM. This compares with IC_50_ values of 18–20 nM for tritiated hypoxanthine isotopic assay and 23–33 nM for *Pf*LDH assay using this strain in our laboratories at Fremantle Hospital in Australia (unpublished observations).

The anti-malarial compounds used in this assay were purchased from Sigma-Aldrich, St Louis, MI, USA (CQ diphosphate), Santa Cruz Biotechnologies, Santa Cruz, CA, USA (pyronaridine (PY) tetraphosphate), Hubei Onward Bio Development Co Ltd, Enshi City, Hubei, China (DHA, artesunate (AS), AM, LM) or kindly donated by Mangalam Pty Ltd, Bangalore, India (PQ phosphate and NQ phosphate). Solutions of 10 mM concentration were prepared for each drug in an appropriate solvent (CQ, PQ and PY in deionized water; AM in methanol; NQ in 50% v/v ethanol; LM in 1:1:1 v/v linoleic acid/Tween 80/ethanol; DHA in 70% v/v ethanol; AS in ethanol). These solutions were further diluted to a stock 1 mM concentration in deionized water. After sterile filtration, stock solutions were aliquoted into airtight microcentrifuge tubes and stored at −20°C. A fresh aliquot was used for each assay.

Red blood cells from slide-positive children were washed three times in standard RPMI 1640-based malaria cell culture medium [17] and, if necessary, diluted to 0.5-1.0% parasitaemia with red blood cells from a malaria-naïve donor of blood type O Rhesus negative. The culture medium consisted of RPMI 1640 HEPES (Sigma Aldrich, St Louis, MO) supplemented with 92.6 mg/L L-glutamine (Sigma Aldrich, St Louis, MO), 500 μg/L gentamicin (Sigma Aldrich, St Louis, MO), 50 mg/L hypoxanthine (Sigma Aldrich, St Louis, MO) and 0.5% w/v Albumax II lipid rich BSA (Life Technologies, Mulgrave, Victoria, Australia) [16]. Drug dilutions were set up in 96-well plates in triplicate, with eight dilutions for each drug. The haematocrit was set at 1% and the liquid volume per well was 200 μL. The assay plates were incubated for 48 hr in a candle jar using the method of Trager and Jensen [18], after which 50 μL of a red cell lysis buffer/Sybr green (Invitrogen, Carlsbad, CA, USA) mixture were added to each well. The plate was incubated for 15 min in the dark. Fluorescence was read on a microplate reader (Fluostar Optima, BMG Labtec, Offenburg, Germany) equipped with a 484 nm excitation filter and a 520 nm absorbance filter.

### Molecular analysis

Parasite isolates where tested for genetic markers associated with drug resistance using a multiplex polymerase chain reaction ligase detection reaction fluorescent microsphere assay (PCR-LDR-FMA) assay as previously described [14,19]. In brief, PCR-LDR-FMA was performed using established primer sequences to detect single nucleotide polymorphisms in the known resistance loci of *pfdhfr* (codons 51, 58, 108, 164)*, pfdhps* (codons 436–437, 540, 581, 613)*, pfcrt* (codons 72–76) and *pfmdr1* (codons 86, 184, 1034, 1042, 1246)*.* Fluorescent products were detected using a Bio-Plex analyzer (Bio-Rad, Hercules, CA, USA). Data analysis was conducted as described previously [20,21].

### Data analysis

Concentrations of anti-malarial drugs for each isolate and anti-malarial were log-transformed and the fluorescence values were normalized such that the smallest value in each dataset represented 0 and the largest value (drug-free control) unity. The dose–response curve *Y* = 100/(1 + 10^*k* (logIC50-log*X*)^) was then fitted to each dataset, where *Y* corresponds to the percentage of growth at drug concentration *X*, and *k* is the Hill slope. For calculations of means and 95% confidence intervals (CI) as well as for analysis of associations between pairs of different anti-malarial drugs, the log_10_ IC_50_ values were used as these were normally distributed by Kolmogorov-Smirnov test. Associations between IC_50_ values were determined using Pearson’s correlation coefficient, and significant pairwise correlations (*P* < 0.05) were considered moderate for 0.3 ≤ r ≤0.50 or strong for r >0.5.

Factor analysis was conducted after testing the cross-correlation matrix for sphericity using Bartlett’s Test and using the Kaiser-Maier-Olkin statistic to determine the appropriateness of the data for this analysis. The distribution of the Eigenvalues of the cross-correlation matrix indicated that factoring into two components was the most appropriate approach. Since it was hypothesized that the underlying factors relate to the mechanisms of drug action and are thus related to each other, a non-orthogonal (direct-oblimin) rotation was applied to the solution.

## Results

### *In vitro* drug susceptibility measurements

**Figure 1:**
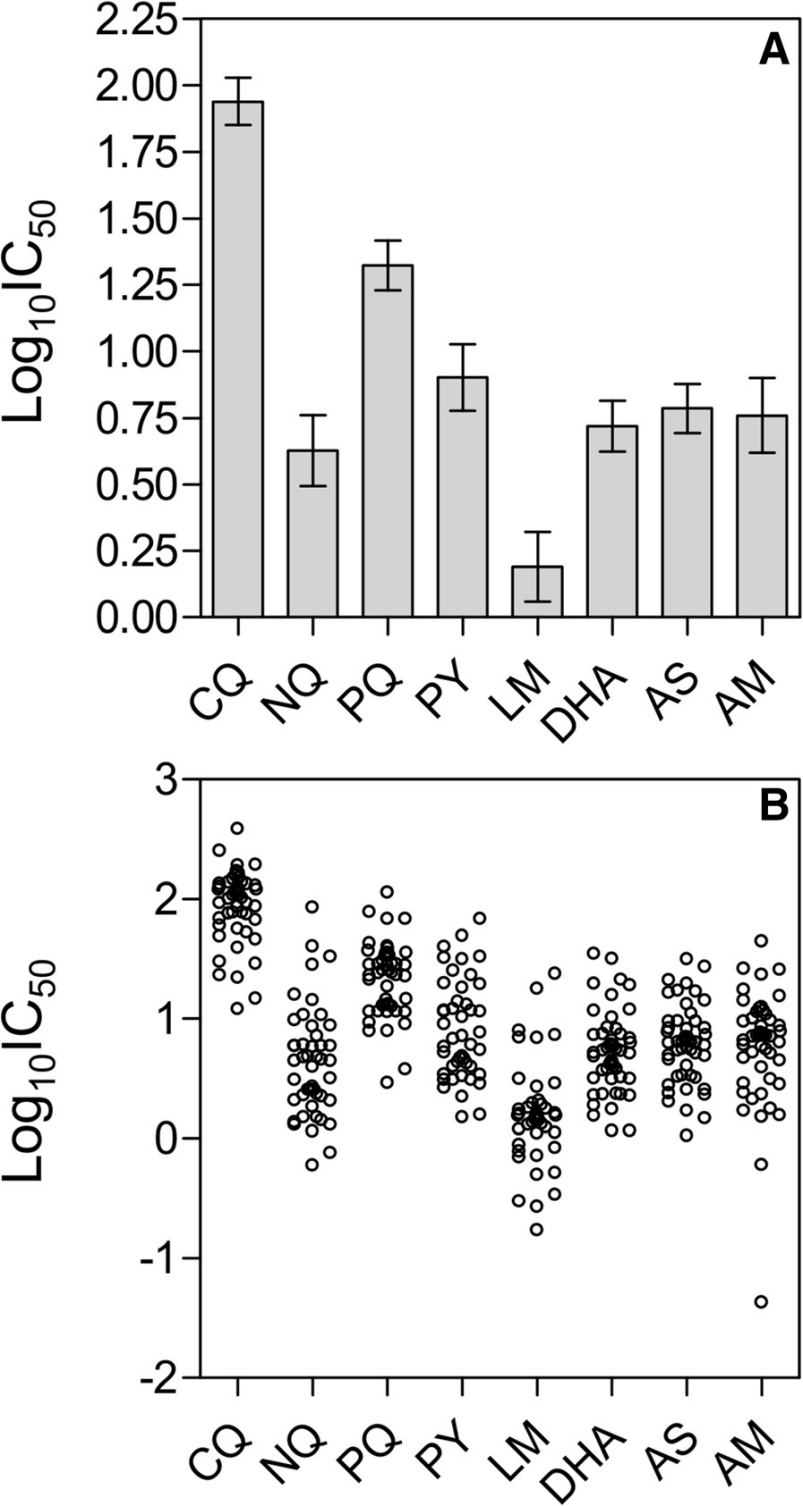
**50% inhibitory concentrations (IC**_50_ **) for the anti-malarial compounds investigated in the present study.** Chloroquine (CQ), piperaquine (PQ), naphthoquine (NQ), pyronaridine (PY), lumefantrine (LM), dihydroartemisinin (DHA), artesunate (AS) artemether (AM). Panel **A** shows the logarithmic means and 95% CIs and Panel **B** shows measurements for single isolates.

From a total of 416 drug assays (52 isolates and 8 drugs), 379 (91.1%) provided a valid dose–response curve that could be used for analysis. Numbers of successful assays per drug were 47 (NQ), 44 (LM), 50 (DHA), 50 (AS), 48 (AT), 45 (PY), 47 (PQ) and 48 (CQ). The growth responses of the parasite isolates to the panel of anti-malarial drugs used in the present study are summarized in Figure 1. CQ resistance (IC_50_ > 100 nM [22,23]) was present in nine out of 48 isolates (19%). For alternative resistance thresholds of 87 nM, 70 nM and 25 nM as recommended by other authors [24-26], the percentages of resistant strains were 26, 42 and 88%, respectively. Although there is no recommended threshold for PQ or NQ, 100 nM has been suggested for PQ and may also be appropriate for NQ [27]. All isolates had an IC_50_ value for PQ and NQ <100 nM, but two strains had IC_50_ values that were close to this value (85 nM for PQ in one case and 96 nM for NQ in the other). Logarithmic mean IC_50_ values and their 95% CIs were 87 (71–107) nM for CQ, 21.0 (16.9-26.1) nM for PQ, 4.2 (3.1-5.8) nM for NQ, 8.0 (6.0-10.6) nM for PY, 1.5 (1.1-2.1) nM for LM, 5.2 (4.2-6.5) nM for DHA, 6.1 (4.9-7.6) nM for AS and 5.7 (4.2-7.9) nM for AM.

**Figure 2:**
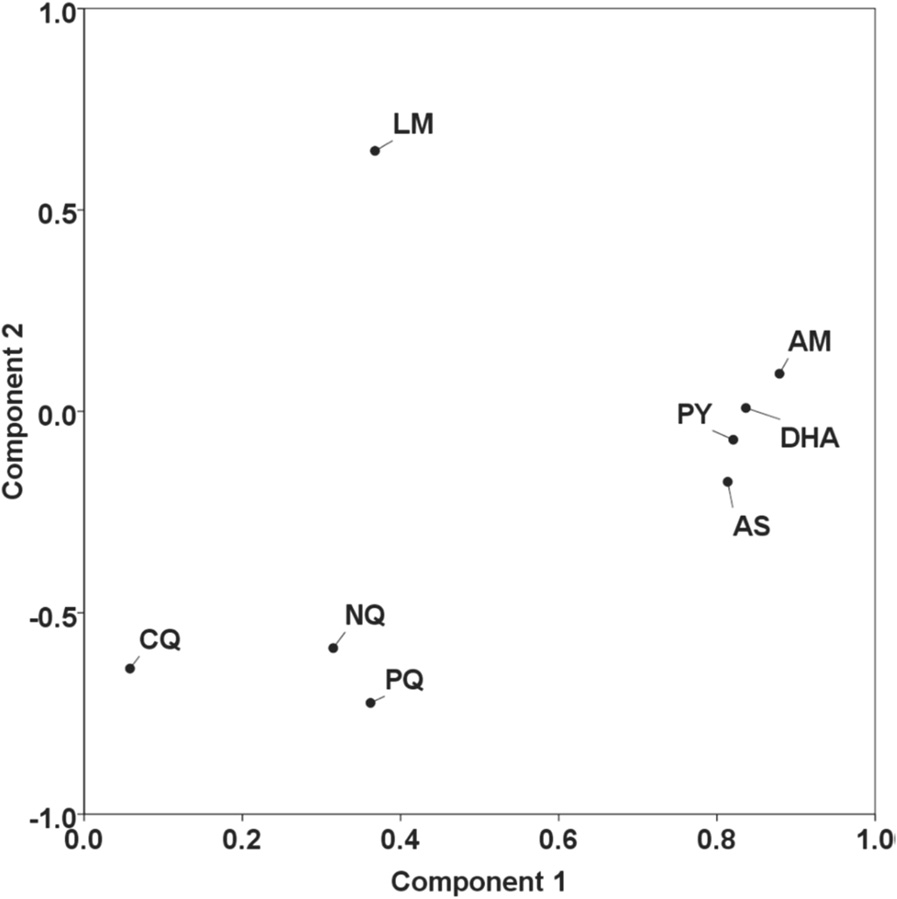
**Factor analysis of IC**_50_ **values.** Kaiser-Maier-Olkin (KMO) statistics and Bartlett’s Test for sphericity indicated the appropriateness of the data for factor analysis (KMO statistic: 0.62, Bartlett’s test p-value: <0.0001). The distribution of the Eigenvalues indicated that a separation into two components was most appropriate. Within the two-component space, three distinct clusters of drugs were observed (1: lumefantrine (LM) 2: chloroquine (CQ), piperaquine (PQ), naphthoquine (NQ); 3: pyronaridine (PY), artesunate (AS), dihydroartemisinin (DHA), artemether (AM)).

Table 1 shows the cross-correlation analysis for each pair of anti-malarial drugs. The strongest correlations were observed between the IC_50_ values for i) CQ, PQ, NQ, and PY (0.31 < r < 0.55) with an exception being that between CQ and PY (*P* > 0.05), and ii) between DHA, AM, AS, and PY (0.45 < r < 0.66) with an exception being that between PY and AM (*P* > 0.05). The IC_50_ values for LM showed no significant correlations with those of any of the other drugs. Factor analysis indicated two underlying components, which may explain most of the variation in drug responses (see Figure 2). Within this two-component space, the eight drugs clustered into three distinct groups (LM on its own; CQ, PQ and NQ; PY, DHA, AM, and AS).

**Table 1:** **Cross-correlation (Pearson’s r) between growth inhibition to anti-malarial drugs where n is the number of paired IC**_50_ **s analysed**

		**Chloroquine**	**Naphthoquine**	**Piperaquine**	**Pyronaridine**	**Lumefantrine**	**Dihydro-artemisinin**	**Artesunate**
Naphthoquine	r	0.35^*^						
	*P*	0.020						
	n	45						
Piperaquine	r	0.44^**^	0.55^**^					
	*P*	0.002	<0.001					
	n	45	44					
Pyronaridine	r	0.19	0.49^**^	0.31^*^				
	*P*	0.23	0.001	0.042				
	n	43	44	43				
Lumefantrine	r	−0.08	0.21	−0.03	0.13			
	*P*	0.62	0.18	0.86	0.40			
	n	42	42	42	42			
Dihydro-artemisinin	r	0.27	0.20	0.33^*^	0.47^**^	0.11		
	*P*	0.065	0.18	0.026	0.001	0.48		
	n	48	47	47	45	44		
Artesunate	r	0.28	0.38^**^	0.27	0.45^**^	0.24	0.66^**^	
	*P*	0.060	0.009	0.067	0.002	0.12	<0.001	
	n	47	46	47	45	44	49	
Artemether	r	0.24	0.37^*^	0.29^*^	0.29	0.04	0.49^**^	0.64^**^
	*P*	0.11	0.012	0.048	0.053	0.78	<0.001	<0.001
	n	46	46	46	45	44	48	48

The data were log-transformed before analysis. The symbols * and ** indicate *P* < 0.05 and *P* < 0.01, respectively.

### Drug resistance markers and their association with *in vitro* drug susceptibility

Most isolates (96%) were monoclonal *pfcrt* K76T mutants of haplotype SVMNT (codons 72 to 76). One isolate contained a mix of CVMNK (wild-type) and SVMNT (mutant). In only one isolate was CVMNK (wild-type) detected as the only clone. This isolate exhibited an IC_50_ of 12.1 nM against CQ, which was the lowest in IC_50_ for CQ found in the study. The polyclonal CVMNK/SVMNT isolate exhibited a CQ IC_50_ of 30.0 nM, which was also amongst the lowest values determined in the present study.

Two mutant haplotypes of *pfmdr1* were identified, the most prevalent being the YYSND type associated with CQ resistance (86% of isolates, N86Y mutation). The NFSND haplotype was found in 5% of isolates (Y184F mutation). The wild-type NYSND occurred in only 9% of isolates. The presence of NYSND was associated with reduced CQ IC_50_ values (*P* < 0.05, see Table 2).

**Table 2:** **Associations between** ***Plasmodium falciparum*** **genetic mutations and IC**_50_ **values for chloroquine (CQ), piperaquine (PQ), naphthoquine (NQ), pyronaridine (PY), lumefantrine (LM), dihydroartemisinin (DHA), artesunate (AS) artemether (AM)**

***pfcrt***	**Wild-type (CVMNK)**	**Mixed**	**Mutant**
CQ	12 (n = 1)	30 (n = 1)	96 (79–117) (n = 39)
NQ	5.9 (n = 1)	0.6 (n = 1)	4.3 (3.9-5.9) (n = 37)
PQ	13.0 (n = 1)	11.4 (n = 1)	21.3 (16.8-27.0) (n = 39)
PY	32.5 (n = 1)	2.2 (n = 1)	7.4 (5.5-9.8) (n = 36)
LM	17.8 (n = 1)	2.0 (n = 1)	1.5 (1.0-2.0) (n = 34)
DHA	6.6 (n = 1)	3.8 (n = 1)	5.0 (3.0-6.4) (n = 40)
AS	6.6 (n = 1))	2.1 (n = 1)	5.4 (3.7-8.0) (n = 38)
AM	6.4 (n = 1)	3.3 (n = 1)	6.3 (5.0-7.8) (n = 39)
***dhps***	**Wild-type (SAKAA)**	**Mixed**	**Mutant**
CQ	84 (59–118) (n = 17)	192 (39–944) (n = 3)	72 (41–124) (n = 8)
NQ	4.6 (2.4-8.8) (n = 16)	7.2 (3.1-16.4) (n = 3)	3.4 (1.7-6.8) (n = 8)
PQ	18.6 (12.4-27.9) (n = 17)	31.9 (19.7-51.7) (n = 3)	17.8 (9.2-34.2) (n = 8)
PY	8.0 (4.7-13.6) (n = 17)	9.0 (3.1-26.3) (n = 3)	7.3 (2.8-18.9) (n = 7)
LM	2.1 (1.0-4.4) (n = 16)	1.5 (1.5-1.6) (n = 3)	1.3 (0.9-2.0) (n = 6)
DHA	4.5 (3.0-6.7) (n = 17)	7.6 (0.8-72.6) (n = 3)	7.0 (3.2-15.1) (n = 8)
AS	6.2 (4.2-9.2) (n = 17)	8.0 (5.6-11.5) (n = 3)	6.9 (2.8-16.9) (n = 8)
AM	6.2 (4.2-9.2) (n = 17)	6.9 (4.7 -10.2) (n = 3)	5.7 (3.4-9.4) (n = 8)
***dhfr***	**Wild-type (NCSI)**	**Mixed** ^**#**^	**Mutant**
CQ	-	161 (22–1195) (n = 3)	89 (71–112) (n = 33)
NQ	-	4.7 (2.0-10.1) (n = 3)	4.1 (2.8-5.9) (n = 32)
PQ	-	17.39 (2.7-113.3) (n = 3)	20.5 (15.9-26.5) (n = 34)
PY	-	3.9 (0.6-27.4) (n = 3)	7.7 (5.5-10.7) (n = 32)
LM	-	1.2 (0.5-2.7) (n = 3)	1.6 (1.0-2.4) (n = 30)
DHA	-	8.0 (0.8-72.3) (n = 3)	4.9 (3.7-6.5) (n = 34)
AS	-	7.4 (4.1-13.4) (n = 3)	5.7 (3.8-8.5) (n = 33)
AM	-	7.5 (2.2-26) (n = 3)	6.2 (4.8-7.9) (n = 34)
***pfmdr1***	**Wild-type (NYSND)**	**Mixed**	**Mutant**
CQ	-	39 (12–125) (n = 3)	96 (77–122) (n = 33)*
NQ	-	1.5 (1.5-1.5) (n = 2)	4.4 (3.1-6.2) (n = 32)
PQ	-	11.5 (6.6-20.1) (n = 3)	20.9 (16.2-27.1) (n = 34)
PY	-	2.2 (0.0-217.1) (n = 2)	7.8 (5.7-10.9) (n = 32)
LM	-	0.8 (0.2-2.8) (n = 3)	1.7 (1.1-2.5) (n = 30)
DHA	-	4.7 (2.9-7.6) (n = 3)	5.1 (3.8-6.8) (n = 34)
AS	-	3.8 (0.5-30.6) (n = 3)	5.8 (3.9-8.7) (n = 33)
AM	-	5.9 (2.5-13.6) (n = 3)	6.4 (5.0-8.2) (n = 34)

Values are given as geometric mean (95% confidence interval [where applicable]) with (number of paired observations).

*P < 0.05 by Mann–Whitney U test; ^#^‘Mixed’ corresponds to a mix of NRNI and NRTI, since no wild types were found.

**Figure 3:**
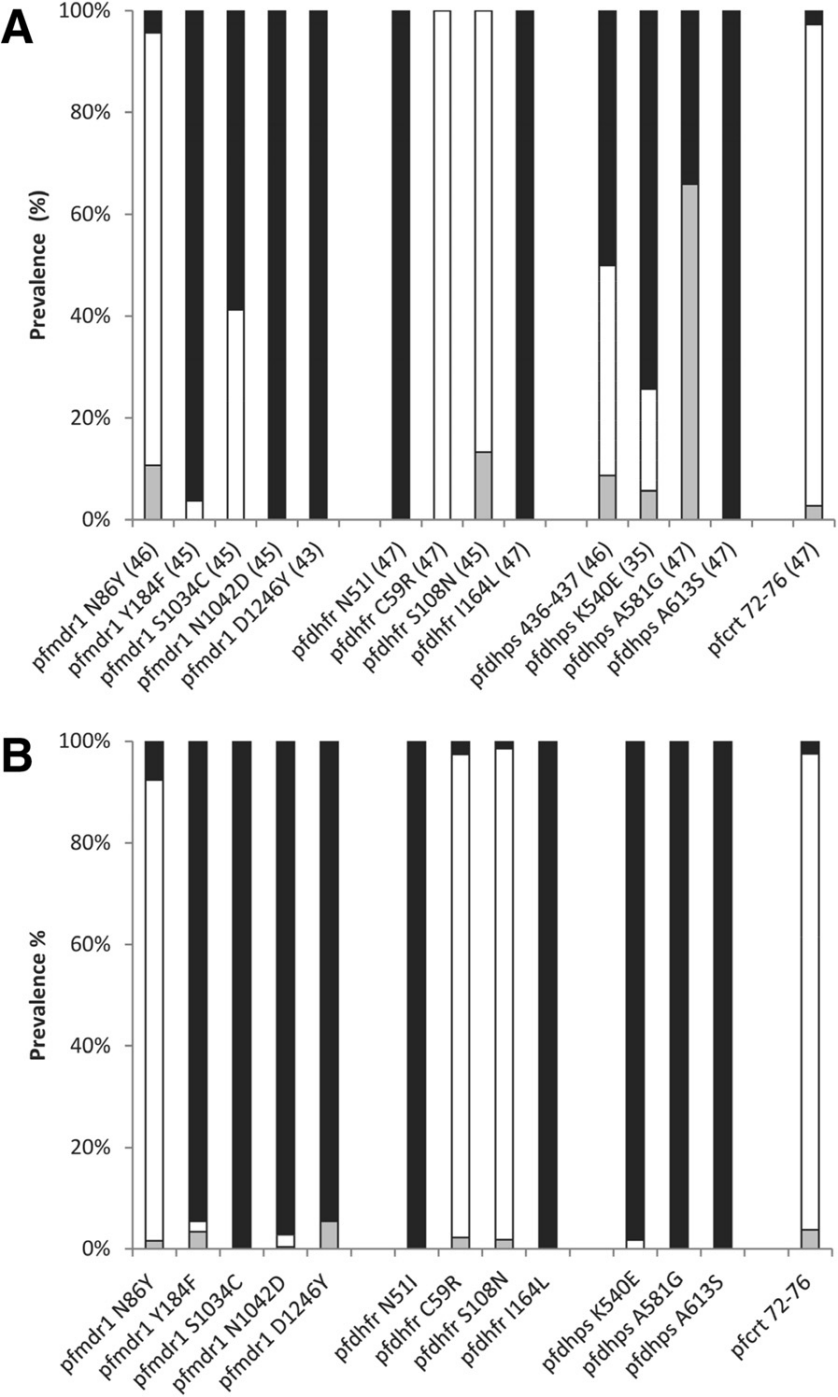
**Prevalence of drug resistance-associated gene mutations in parasite isolates from north coastal PNG.** Numbers in parentheses denote the number of samples that resulted in interpretable genotyping results. **Panel A** shows data collected in the present study and **panel B** shows data collected between 2005 and 2007 [14]. Percentages of wild-type genotypes (■), mutants (□) and mixed infections (grey square) are shown.

No wild-type *pfdhfr* was found in the isolates studied. All isolates carried the C59R mutation and the S108N mutation (with absence of mutation at codons 51 and 164, Figure 3). Among them, 13% of isolates were polyclonal with the S108T mutation detected as well (haplotype NRTI). The SAKAA wild-type haplotype of the *pfdhps* gene was found as the only haplotype in 60% of all isolates and in a further 9% in polyclonal infections. The SGKAA and the SGEAA mutant haplotypes occurred in 9 and 14% as monoclonal infections, respectively, and in a further 9% of polyclonal infections. An additional haplotype FGEAA was found in 6% of infections, mixed with the SGEAA haplotype.

### Comparison of the present findings with those of similar surveillance in 2005–2007

The geometric mean and 95% CIs for parasite isolates obtained from children with uncomplicated malaria in a study carried out in Madang and East Sepik Provinces between 2005 and 2007, as well as the present drug susceptibility data, are shown in Table 3 [13]. The method used in the earlier study was the *Pf*LDH assay [28]. The IC_50_ values were lower for the present isolates than those obtained an average of six years earlier for CQ, NQ and LM, but higher for PQ and DHA.

**Table 3:** **Comparison of IC**_50_ **values determined using the** ***Pf*** **LDH assay in 2006 in the same population and the Sybr Green assays in 2012**

	**2005-2007**		**2011-2013**		***P*** **-value**
**Drug**	**n**	**IC**_**50**_	**n**	**IC**_**50**_	
Chloroquine	63	167 (141–197)	48	87 (71–106)	<0.001
Piperaquine	57	11.7 (10.2-13.4)	47	21.1 (17–26)	<0.001
Naphthoquine	41	7.0 (5.5-8.8)	44	4.2 (3.1-5.8)	0.015
Lumefantrine	25	2.4 (1.8-3.1)	47	1.5 (1.1-2.1)	0.075
Dihydroartemisinin	30	2.1 (1.5-2.9)	50	5.2 (4.2-6.5)	<0.001

Data are geometric mean and (95% CI) and *P*-values are two-tailed from Student’s *t*-test.

The *P. falciparum* polymorphisms associated with anti-malarial drug resistance for the isolates obtained between 2005 and 2007 [14], as well as those of the present study, are shown graphically in Figure 3. There had been no change in the near-fixation of *pfcrt* K76T in the six years between studies and the prevalence of *pfmdr1* mutations was also similar. The NRTI haplotype was not reported in the previous study using the same methodology [14] but it was detected by the present genotyping. The *pfdhps* wild-type gene (SAKAA) was found as the only haplotype in 60% of all isolates and in a further 9% of polyclonal infections, a lower prevalence than the ~100% reported previously [14].

## Discussion

The present data demonstrate that there have been changes in the drug resistance characteristics of parasite isolates collected between 2005–2007 and 2011–2013 from areas of north coastal PNG with intense malaria transmission. Although a different methodology was used to assess *in vitro* sensitivity in the present study, and notwithstanding limitations in assigning thresholds for *in vitro* drug sensitivity [29], more strains appeared CQ-sensitive than in 2005–2007 [13,14] despite the majority retaining the mutant *pfcrt* K76T allele over time. There were also apparent temporal reductions in the IC_50_s of LM and NQ, while those for PQ and DHA increased albeit still within relatively low nM ranges. The proportion of parasites carrying the wild-type *pfdhps* gene had fallen over time and more mutations had appeared in *pfdhps* in the 2011–2013 isolates, consistent with continued use of SP in the study area. Factor analysis suggested that the *in vitro* susceptibilities of PNG *P. falciparum* strains to LM and PY may be unrelated to those of other long half-life ACT partner drugs, with the IC_50_ of PY clustering with those of the artemisinin derivatives. Interpretation of the present and previous data needs to take into account several factors. These comprise i) temporal changes in anti-malarial drug use in the study areas, ii) potential effects of the introduction of non-pharmacological strategies to reduce malaria transmission, and iii) differences in assay methodology between 2005–2007 and 2011–2013.

Recommendations regarding replacement of regimens based on CQ and SP by ACT for treatment of uncomplicated malaria in PNG children were implemented in 2010 [6], but translation of this policy into practice has been slow. In addition, the use of CQ and SP as first-line intermittent preventive therapy (IPT) in pregnancy has continued, and an IPT trial in infants involving SP was conducted in the Mugil area (from where most of the present isolates were collected) between 2006 and 2010 [30]. Therefore, CQ-SP drug pressure had been reduced, but not eliminated, over a period of two to three years leading up to isolate collection in the study area.

The dynamics governing repopulation by CQ-sensitive strains in areas in which CQ treatment pressure has been removed completely are not well understood, but the time-scale is probably approaching a decade [24,31,32]. The fact that the present isolates were collected after a short period of incomplete removal of CQ-SP pressure is reflected in the present molecular analyses which showed no reduction or an increase in parasites carrying genetic markers that correlate with CQ-SP resistance. However, CQ resistance mutations are frequently found in isolates that show *in vitro* susceptibility [24,25,33], and there is also evidence that CQ IC_50_ values can fall relatively quickly (within a few years) after reduction in drug pressure [34,35].

The introduction of long-lasting insecticide-impregnated bed nets (LLINs), such as was started on a large scale in PNG in 2004 [36], could theoretically also attenuate drug pressure by reducing malaria transmission. The evidence for this effect on molecular resistance markers in studies from sub-Saharan Africa is conflicting [37,38]. However, these studies were relatively short-term compared with the time needed for re-establishment of full sensitivity after drug withdrawal [24,31,32] and no *in vitro* susceptibility data were presented. It remains possible that increasing LLIN use in coastal PNG between 2005–2007 and 2011–2013, together with the partial replacement of CQ-SP by ACT, both contributed to the lower IC_50_ for CQ in the present study.

There is no accepted standardized protocol for determining *in vitro* anti-malarial drug susceptibilities and the results may differ according to the methodology employed. There is evidence that the *Pf*LDH assay generates higher IC_50_ values than other methodologies including the Sybr Green assay used in the present study [16,39,40]. However, the reported differences are typically modest (typically 10–30 nM across a range of IC_50_ values, as seen with our own data for the 3D7 strain) compared with the substantial reduction in CQ IC_50_ observed between 2005–2007 and 2011–2013. The increase in PQ IC_50_ over time in north coastal PNG might appear paradoxical given than CQ and PQ susceptibility have both been considered to reflect *pfcrt* mutations [41]. Nevertheless, not all studies show this relationship [42], while the PQ IC_50_ values in both time periods were both well below the conventional 100 nM cut-point for resistance in all but one isolate in the present study. The small temporal increase in DHA IC_50_ may also be of no clinical significance given that the IC_50_ value in all isolates was in the very low nM range. Standard drug susceptibility assays do not detect early stage artemisinin resistance defined by a slow parasite clearance time for which there is now a molecular marker [43], but the isolates were obtained between 2011 and 2013 from patients in a clinical trial [15] in which there was no evidence of longer parasite clearance times after AM-LM than in the equivalent trial conducted from 2005 to 2007 [4].

Several studies have shown moderate to strong correlations between *in vitro* parasite responses to CQ and PY [44,45] while in others, including the present study, there has been no such association [46,47]. The future of PY-containing ACT is uncertain because of hepatotoxicity [48]. However, it does not appear to exhibit cross-resistance with CQ in the present parasite isolates, which would be an advantage if PY-based ACT became available for repeated use in PNG. The present data confirm the moderate associations between CQ, PQ and NQ which were also observed previously in north coastal PNG [13]. Although the IC_50_s for the latter two compounds are relatively low, their significant association with CQ susceptibility may have implications for the longevity of ACT formulations incorporating them. The lack of clustering of LM susceptibility with other longer half-life anti-malarial drugs suggests that it may have an independent mechanism of action. As has been done by other groups [49-51], we included the three artemisinin drugs in common clinical use, even though DHA is the active metabolite of both AS and AM *in vivo*, since there is evidence that their activity against *P. falciparum in vitro* is not uniform [52]. Consistent with this latter observation, the association between AM and DHA was only moderate while those between AS and both AM and DHA were the strongest observed.

## Conclusion

Although the prevalence of molecular markers of anti-malarial drug resistance has not fallen in north coastal PNG over the six years between the present study and a previous cross-sectional survey, CQ susceptibility has increased even allowing for different methods of *in vitro* parasite drug sensitivity testing. This may reflect attenuation of drug pressure through changes in national treatment policy and the roll-out of LLINs in the study areas. Although there are no parasite strains showing definite *in vitro* resistance to PQ and NQ, the association of PQ and CQ IC_50_s suggests that future susceptibility testing should include these ACT partner drugs which are currently available in PNG as alternatives to LM. Since LM drug susceptibility appears independent of other available and potential ACT partner drugs, the use of AM-LM as first-line treatment of uncomplicated malaria in PNG may not lead to clinically significant cross-resistance.

## Abbreviations

ACT: Artemisinin combination therapy
AM: Artemether
AS: Artesunate
CI: Confidence interval
CQ: Chloroquine
DHA: Dihydroartemisinin
IC_50_: *in vitro* concentration required for 50% parasite growth inhibition
IPT: Intermittent preventive therapy
LLIN: Long-lasting insecticide-impregnated bed net
LM: Lumefantrine
NQ: Naphthoquine
PCR-LDR-FMA: Multiplex polymerase chain reaction ligase detection reaction fluorescent microsphere assay
PNG: Papua New Guinea
PQ: Piperaquine
PY: Pyronaridine
SP: Sulphadoxine-pyrimethamine
